# Perceptions on wearable sensor-based interventions for monitoring of opioid therapy: A qualitative study

**DOI:** 10.3389/fdgth.2022.969642

**Published:** 2022-10-21

**Authors:** Brittany P. Chapman, Evan Lucey, Edward W. Boyer, Kavita M. Babu, David Smelson, Stephanie Carreiro

**Affiliations:** ^1^ Department of Emergency Medicine, Division of Medical Toxicology, Tox(IN)novation Lab, UMass Chan Medical School, Worcester, MA, United States; ^2^ Department of Emergency Medicine, The Ohio State University, Columbus, OH, United States; ^3^ Department of Psychiatry, Division of Addiction Psychiatry, UMass Chan Medical School, Worcester, MA, United States

**Keywords:** digital health, opioid, wearable, mHealth, perceptions

## Abstract

Prescription opioid use is a risk factor for the development of opioid use disorder. Digital solutions, including wearable sensors, represent a promising opportunity for health monitoring, risk stratification and harm reduction in this treatment space. However, data on their usability and acceptability in individuals using opioids is limited. To address this gap, factors that impact usability and acceptability of wearable sensor-based opioid detection were qualitatively studied in participants enrolled in a wearable sensor-based opioid monitoring research study. At the conclusion of the monitoring period, participants were invited to take part in semi-structured interviews developed based on the technology acceptance model. Thematic analysis was conducted first using deductive, then inductive coding strategies. Forty-four participants completed the interview; approximately half were female. Major emergent themes include sensor usability, change in behavior and thought process related to sensor use, perceived usefulness in sensor-based monitoring, and willingness to have opioid use patterns monitored. Overall acceptance for sensor-based monitoring was high. Aesthetics, simplicity, and seamless functioning were all reported as key to usability. Perceived behavior changes related to monitoring were infrequent while perceived usefulness in monitoring was frequently projected onto others, requiring careful consideration regarding intervention development and targeting. Specifically, care must be taken to avoid stigma associated with opioid use and implied misuse. The design of sensor systems targeted for opioid use must also consider the physical, social, and cognitive alterations inherent in the respective disease processes compared to routine daily life.

## Introduction

Opioids are powerful analgesics that have been used to alleviate human suffering for hundreds of years; they are also at the center of a modern-day public health crisis that claims the lives over 100 people daily in the United States alone. The early 2000's marked a turning point in opioid-assisted pain management. New standards enacted by The Joint Commission (TJC) and the “Pain as a Fifth Vital Sign” campaign pressured physicians to meet strict pain management metrics in patients (1). Heightened scrutiny of patient pain and pain alleviation resulted in aggressive opioid prescription practices. Between 2000 and 2014, opioid consumption in the United States more than tripled (2) with a peak opioid dispensing rate in 2012 at 81.3 prescriptions per 100 Americans (3). Prescription opioid medications have been correlated with increased risk rates of opioid use disorder (OUD), morbidity, and mortality (4–6), resulting in public health, legislative and clinical policies aimed at reducing opioid prescribing. Further complicating the issue is the current widespread availability of potent synthetic opioids, namely fentanyl and its analogues, which have surpassed prescription opioids as a major contributor to the opioid crisis in the last decade. The Centers for Disease Control and Prevention (CDC) describe the opioid crisis death pattern over the last twenty years occurring in three waves: Wave one (1999–2010) characterized by a sharp increase in prescription opioid deaths, wave two (2010–2013) characterized by a sharp increase in deaths from heroin, and wave three (2013-prsent) characterized by a rapid increase in death from synthetic opioids (7).

Despite greater awareness for the associated risks with prescription opioids, improved prescription practices, and the majority of current opioid-related mortality being attributable to synthetic opioids, prescription opioid-related overdose deaths continue to be a significant problem. In fact, the number of prescription-drug deaths remains relatively steady despite widespread efforts to curb prescribing, with 17,029 deaths in 2017 and 16, 416 deaths in 2020 (compared to 3,442 deaths in 1999) (8). Furthermore, some clinicians raise concern that restrictive opioid prescribing practices hinder access to opioids for patients who truly need them, paradoxically causing them to turn to illicit sources to self-treat pain (9, 10). A small, but significant percentage of patients treated with opioid analgesics will progress to misuse, addiction, and/or overdose. Current clinical strategies are limited in their ability to predict which patients are at risk and which patients have already progressed to more problematic use. With the well-documented risks associated with prescribed opioids, novel strategies that prevent adverse opioid-related outcomes in the clinical setting must be explored.

Mobile health (mHealth) is a rapidly developing field that employs mobile technologies to facilitate or enhance patient care. Functionality offered by wearable sensors, mobile phones, and interactive electronic applications allow for continuous remote data collection and patient monitoring, providing clinicians additional objective and/or qualitative data to modify treatment plans. Wearable technologies come in many form factors including but not limited to wrist-worn sensors, cutaneous patches, or textiles (e.g., vests or arm bands). Heart rate, electrodermal activity, and triaxial locomotion are a few examples of objective measurements that can be obtained passively by wearable sensors. Active measures, including surveys and other tasks, obtain patient-reported data that can complement and contextualize objective measurements. These data can be collected and stored with minimal burden on the end-user or clinician. Integrating mHealth technologies with cloud-based systems further expands capabilities by making real-time (or near real-time) data review possible. These technologies could provide a proactive approach to increase prescription opioid analgesic safety by allowing clinicians to monitor patients for adverse outcomes in the outpatient setting- for example objectively documenting medication use and adherence patterns, early identification of opioid dependance, and monitoring for opioid overdose.

Exploration of mHealth as a supporting tool in the treatment of substance use disorders (SUDs), including opioid use disorder is well underway (11, 12). mHealth can be used to monitor patients receiving prescription opioid medications using a combination of objective and patient-reported assessments. Other approaches have described the use of digital health tools to facilitate harm reduction, including opioid overdose detection and automatic delivery of the antidote naloxone (13, 14). For example, Chan et al. evaluated the computational and mechanical functionality of a novel closed-loop, on-body sensor designed to automatically inject naloxone after detecting an opioid overdose (15).

Although physiologic and computational aspects of wearable sensor-based opioid detection are critical to ensuring feasibility and accuracy, user experience is equally, if not more so, important for the success of interventions requiring active participation on the part of the user. End-user experience evaluations provide rich information about the target population's perceptions on factors that impact usability, facilitators and barriers to uptake, and acceptability. Ultimately, a perfectly accurate mHealth intervention will be rendered useless if the intended end-users fail to engage with it. Mobile health interventions provide unique opportunities for patients to interact with clinicians and engage in their own healthcare; the potential impacts of these interventions too often go unrealized because of low engagement (16, 17). The World Health Organization (WHO) has identified user experience as an important component in the design and evaluation of digital health intervention (18), and a user-centered design process is increasingly being recognized as the gold standard in mHealth (16, 17, 19). Existing literature supports the notion that digital health products (and monitoring) are appealing and acceptable to patients with various health conditions, including mental health diagnoses, diabetes, and heart failure (20–22). Numerous individual-level factors are known to impact the acceptability of wearable sensor-based health interventions, including cognitive ability, social characteristics, technical knowledge, and cultural norms (23–27). Within the SUD treatment space, our work and other investigators have found generally high acceptability among individuals who are actively using drugs (including cocaine and opioids) and those in recovery from SUDs (28–30). However understanding the specific factors that impact usability and create barriers to use will be critical: user experience, specifically stigma and device discreetness need to be evaluated in the target populations under unsupervised conditions to determine feasibility of device deployment.

There is growing support that mHealth-based tools such as mobile applications (“apps”), Short Message Service (SMS) text-based interventions, and wearable sensors are acceptable among patients who use prescribed opioids in the setting of chronic pain to help facilitate opioid tapering (31) and monitor physical activity as a complementary therapy (32). However, there is a relative paucity of literature on the acceptability of digital health interventions to specifically monitor opioid intake upon initiation of opioid therapy, which is a currently underexplored opportunity for digital health interventions to facilitate safer opioid use. To address this gap, this study aims to: (a) explore participant perceptions on the use of wearable sensors to monitor prescription opioid use; (b) identify barriers and facilitators to adopting this technology for this indication; and (c) Compare perceptions by sex and opioid use history.

## Methods

### General study overview

This is a qualitative analysis of data obtained as part of a larger observational study of adults presenting to a tertiary care academic medical center in Worcester, Massachusetts, who were prescribed opioid analgesics. The purpose of the parent study was to use physiologic data from a wearable sensor to detect opioid use and identify early signs of opioid dependence. Analysis of the wearable sensor data is presented elsewhere (33).

**Figure 1:**
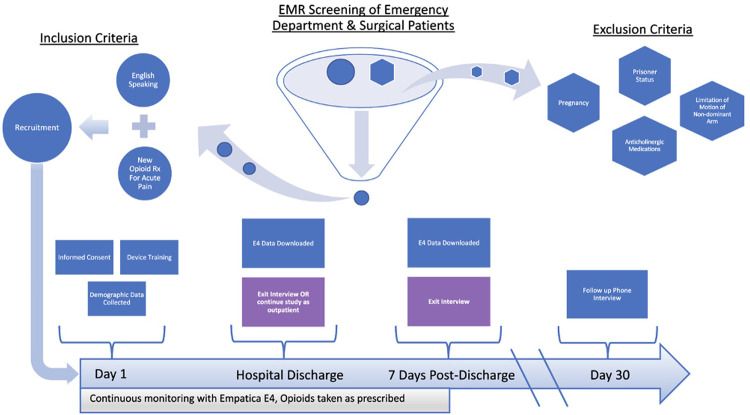
Screening process and study flow. EMR, electronic medical record; Rx, Prescription.

**Figure 2:**
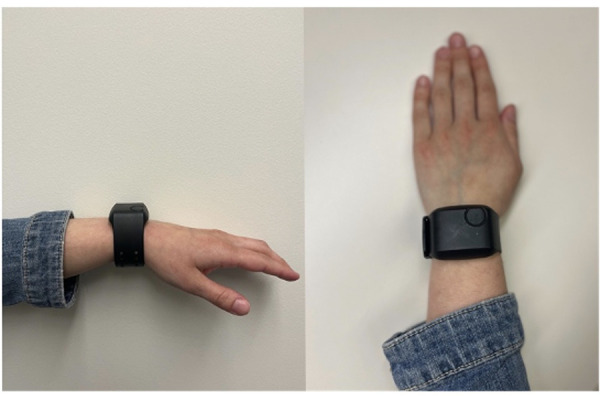
Empatica E4 secured to adult female wrist.

All study-related protocols were reviewed and approved by the UMass Chan Medical School Institutional Review Board and informed consent was obtained from all study participants. Figure 1 displays the screening process and flow of participants through the parent study. Participants were recruited from the emergency department (ED) and surgical clinic settings of an urban, tertiary care academic medical center. Following informed consent, study staff provided a brief training on the use of the device, and then instructed participants to wear the E4 (Empatica, Milan, Italy, Figure 2) on their non-dominant wrist at all times while in the hospital and during waking hours once discharged (34). Physiologic data (heart rate, heart rate variability, triaxial accelerometry, and skin temperature) were acquired continuously throughout their hospitalization and for up to seven days post-hospital discharge. Upon completing the protocol (either upon hospital discharge or up to seven days post-discharge), participants were interviewed regarding their experiences taking opioid analgesics and using the sensor. The goal of these interviews was to elicit formative information regarding attitudes/beliefs towards wearable sensors in the context of opioid use monitoring. Recruitment for the study ceased once trends of data redundancy were observed and new themes ceased to emerge, indicating thematic saturation.

### Inclusion and exclusion criteria

Individuals were considered eligible for the study if they met the following criteria: (1) age 18 years or older; (2) being treated for acute pain with opioid analgesics; (3) able to speak and read in English; and (4) willing and able to provide informed consent. Individuals were excluded from participation if they met the following criteria: (1) significant limitation of motion of the non-dominant arm (i.e., fracture, cast, amputation); (2) known pregnancy; or (3) prisoner status.

### Baseline data collection

Demographic and historical data, including sex, race, ethnicity, medical history, opioid use history, and substance use history, were collected from electronic medical records using a standardized data abstraction form then verbally confirmed with participants. Opioid use history was used to stratify participants into one of three groups: naïve, chronic, or occasional. Those who did not have opioids prescribed to them within the six months prior to study enrollment and did not have a history of opioid use disorder were classified as naïve. Participants classified as having a history of chronic were: (1) opioid use were either maintained on provider-prescribed opioids (i.e., for chronic pain) at baseline; (2) the time of study enrollment actively using non-prescribed opioids, and/or (3) had a history of OUD with less than five years of sustained recovery. The occasional opioid use class included participants that did not meet strict definitions for either the naïve or chronic use groups, but shared qualities of both.

### Qualitative data collection

Semi-structured qualitative interviews were conducted during study exit visits. An interview guide (see Supplementary Material) grounded in technology acceptance model (TAM) principles (35) was developed to ensure key topics were addressed. The TAM explores themes of perceived usefulness, ease of use, acceptance, and actual usage as specific human factors that influence the degree individuals will sustain their use of novel technology. This guide was not a rigid script and therefore allowed study staff the flexibility to adapt and clarify questions to suit the needs of each participant and pursue any relevant, emerging themes as they evolved. Audio from the interviews was digitally recorded and later transcribed verbatim by a trained medical transcriptionist.

### Data analysis

Thematic analysis was conducted to code and analyze qualitative data from semi-structured exit interviews. The coding structure was initially developed based on deductive codes from the interview guide. Inductive codes were then derived from a review of the interview transcripts themselves. Once the coding scheme was developed, two coders were identically trained to employ the coding scheme prior to independently coding each transcript. The coders were blinded from participant demographics and historical data during the coding process to control for observer bias. Despite masking this information, instances where participants revealed demographically or historically identifying information could not be completely controlled given the semi-structured interview design. The two coded transcripts were then compared to resolve interobserver discrepancies and ensure comprehensive, consistent coding. In cases where a consensus was not reached between the two coders, a team discussion was initiated, and a joint decision was made. The principal investigator of the study was excluded from the coding and transcript review processes. The final agreed-upon codes were entered into NVivo qualitative data analysis software (QSR International Party Limited, Melbourne, Australia) which was used to explore and evaluate relationships between the coded data and individual participant attributes (36). The NVivo framework matrix analysis feature was used to stratify coded interview data by biologic sex (male and female) and opioid use history (naive, chronic, and occasional). Comparative analysis was done while reviewing transcripts extracted by participant classification and five themes of interest.

## Results

### Sample characteristics

Of the *N* = 60 participants recruited for the parent study from August 2016 to October 2020, 44 completed a semi-structured exit interview. The final cohort of included participants was 50% female, with a mean age of 47 years. Sixty percent were opioid-naive at the time of study enrollment, and 21% had a history of chronic opioid use. Detailed demographics of the study population, including characteristics of the participants from the parent study who did not complete the exit interview, are listed in Table 1. Individuals who did not complete the interview were more likely to be male, more likely to have a SUD, and were more likely to use opioids chronically.

**Table 1:** Demographic data of parent study participants (*N* = 60) who completed the qualitative interview (responders; *N* = 44) and those who declined to participate (non-responders; *N* = 16).

	Responders (*N* = 44)	Non-Responders (*N* = 16)
Age (in years)		
Mean (SD)	47.0 (14.6)	43.0 (14.7)
Range	16.0–77.0	20.0–79.0
Sex		
Male	22 (50.0%)	10 (62.5%)
Female	22 (50.0%)	6 (37.5%)
Race		
American Indian or Alaskan Native	2 (4.5%)	0 (0.0%)
Black or African American	2 (4.5%)	1 (7.1%)
White	36 (81.8%)	13 (92.9%)
Hispanic/Latinx	4 (9.1%)	4 (25.0%)
Opioid use class		
Naive	28 (63.6%)	7 (43.8%)
Occasional	7 (15.9%)	2 (12.5%)
Chronic	9 (20.5%)	7 (43.8%)
Chronic Pain Syndrome	14 (31.8%)	6 (37.5%)
Psychiatric history	25 (56.8%)	13 (81.2%)
Lifetime history of SUD	17 (38.6%)	11 (78.6%)
Lifetime history of IVDU	1 (2.3%)	3 (23.1%)

*N*, sample size; SD, standard deviation; SUD, substance use disorder; IVDU, intravenous drug use.

### Thematic analysis

The qualitative analysis revealed four major themes related to sensor use: usability, change in behavior and thought process, perceived usefulness in monitoring, and willingness to have their opioid use patterns/responses monitored by their provider. Exemplar quotes for each theme are outlined in Table 2. A framework matrix of themes by sex and opioid use history is presented in Table 3.

**Table 2:** Exemplar participant quotes from the five main study themes.

**Sensor Usability**
“[Using the sensor] is so easy. It's just like a watch.”
- 41-year-old male with a history of occasional opioid use
“It was no different than wearing a FitBit except if you used the medicine you had to push the button, and I think that is fairly easy. If you open up a pill bottle you just push the button at the same time.”
- 48-year-old opioid naïve female
**Change in Behavior and Thought Process Related to Sensor Use**
“Basically [the sensor] makes you more aware when you’re taking [the opioid] and aware of paying attention to how it is reacting with your body”
- 31-year-old opioid naïve female
“I actually think wearing the sensor made [taking the opioid pain medication] more controlled. Because I felt like someone was watching me the whole time.”
- 35-year-old male with a history of occasional opioid use
“[By wearing the device and being part of the research] I felt more welcome at the hospital because people are more concerned about the medications and how I’m doing. Felt more like a home.”
- 41-year-old male with a history of occasional opioid use
Perceived Usefulness in Sensor-based Monitoring*
“Actually yeah, I would wear the sensor in the future to monitor opioid use, I think it's good because you know, you have that sensor make you more aware … I actually felt like wearing the sensor made me take the pain medication more controlled. Because I felt like someone was watching me the whole time.”
- 35-year-old male with history of occasional opioid use
“[The sensor would be a good tool for doctors so] they can control where the pain is coming on, like the time of day all that, and kinda base dosages [of pain medication] off of that, too.”
- 31-year-old opioid naïve female
“I think [the sensor] would [help me keep track of my opioid use patterns]. I think you would have that ‘hey, I’m pushing this button, maybe I’m using a little too much [opioid pain medication], or should I – do I really need it?’ Might think twice before using.”
- 48-year-old opioid naïve female
**Willingness to be Monitored and Desired Features in a Sensor-based System**
“[I’d be willing to wear the sensor] for as long as they wanted me to; it doesn’t bother me so if I can help somebody, why not.”
- 71-year-old female with a history of chronic opioid use
“[Wearing two sensors] would be too much.”
- 22-year-old opioid naïve male
“ … The sensor is straight black so I figure like some colors might make it more fun … ”
- 31-year-old female opioid naïve female
“I’d be willing to wear the sensor again for the same indication in the future. Hoping and praying it's been redesigned by then’
- 53 year-old opioid naïve male

**Table 3:** Framework matrix table of themes by sex and opioid use history (naïve, chronic, occasional).

	Sex	Opioid use history
Sensor usability	A majority of both groups felt the sensor was unnoticeable while wearing, easy to use and comfortable Subset of females were more likely to have negative opinions of the aesthetics (e.g. size and bulk), experience difficulty with proper wristband sizing, and cite the lack of customizability as barriers for continuous use	All groups described the device to be easy to use and comfortable Sensor aesthetics, size, and lack of functionality were the most significant barriers within the naïve opioid use class Amongst chronic and occasional opioid use classes, mechanical specifications, such as short battery life and charge frequency, were more commonly cited barriers for use
Change in behavior and thought process related to sensor use	Other than minor changes in daily routines in a minority of participants, there were no major changes in behavior while wearing the sensor amongst the female and male groups A majority of participants from both groups reported no major changes in thoughts or behavior process while wearing the sensor	Other than minor changes in daily routines in a minority of participants, there were no major changes in behavior amongst the three opioid use history classes Opioid naïve participants did not report any significant change in thought process while wearing the sensor Some participants from the chronic and occasional opioid use history classes related feeling more prioritized and cared for because of the monitoring Heighted awareness to opioid medication consumption and the medications effects were exclusively reported by members of the occasional opioid use history class
Perceived usefulness in sensor-based monitoring	Female and male participants shared a similar opinion that the device would be useful for monitoring opioid medication use	No difference noted among groups^a^
Willingness to be monitored and desired features in sensor-based systems	No difference in length of time participants would be willing to wear the device, and unwillingness to wear a sensor on both wrists Both groups suggested adding an interactive screen the sensor with varying degrees of additional sensor functionality (e.g., step count, heart rate monitoring, clock). The most common suggested improvement was a clock to aid with medication dosing intervals Females more frequently suggested improvements to device aesthetics and material composition.	No difference in length of time participants would be willing to wear the device beyond the standard length of the study Participants from the chronic opioid use history class were more willing to wear two sensors simultaneously The naïve use class focused suggestions on device aesthetics and functionality unrelated to opioid monitoring Chronic and occasional use classes were more likely to recommend improvements to the device's mechanical specifications

^a^ Perception of usefulness were predominantly discussed by opioid naïve class participants, limiting the sample size for chronic and occasional use class participants for this category.

#### Sensor usability

Usability was a central theme and it dominated discussions regarding the sensor. Overall, participants were either indifferent (e.g., did not notice it, felt like it blended in with everyday life) or enjoyed wearing it. No participants expressed strong feelings of dislike toward the device. A major facilitator of use was overall familiarity with off-the-shelf wrist-worn sensors. The E4 was commonly compared to a conventional watch or smartwatch, and the wrist-based location felt familiar and “natural.” The short duration of use in the context of this study (i.e., for 1–2 weeks) was also considered a reasonable duration.

Major barriers to usability included complexity, technical difficulties, and aesthetics. Although the device itself is not particularly complex to operate, it was not considered intuitive by participants. For example, flashing lights on the current model were confusing as participants were not always sure what the light indicated. Training materials distributed to participants included a graphic key to explain the notifications; however, participants considered it cumbersome to find the pamphlet and look up the meanings. Participants expressed the desire for intuitive, on-device status indicators (preferably a screen) that would not require training or reference material to understand. This perceived complexity may have been compounded by the acuity of the participant's medical condition and/or medication-induced transient alterations in mental status. One participant reported that when they were in severe pain and/or under the influence of mind-altering medications, they did not have the mental capacity (or patience) to learn how to use the device.

Technical challenges were also not well-received and were regarded as inconvenient. Participants expressed higher expectations for a wearable device used for healthcare compared to those used for recreational and sporting activities. They also expected the device to function smoothly without significant troubleshooting on their end. Frequent charging was considered a barrier; the expectation was that it would perform at least at the standard of current commercially available devices (i.e., not requiring daily charging).

Aesthetics were cited as very important, and device size was the most frequently commented on factor, both in this regard and in terms of comfort. Participants specifically cited the “bulky” nature of the sensor (which was occasionally associated with court-ordered monitoring devices) and preferred a low-profile design. Commercial sensors (e.g., Fitbit, Apple Watch) were frequently mentioned either to be comparable to the E4 or something they would prefer to wear. Additionally, ease of physical use (e.g., putting the device on, connecting it to the charger) was an important factor and commonly cited as needing to be more effortless. Lack of customizability was identified as a barrier and an area where if improved, would increase usability substantially. For example, the sensor band had limited sizing options. The lack of adjustability made participants (particularly those with larger wrists) feel like they were not considered during the design process. The types of preferred materials and sensor styles differed among participants; some were concerned about sweating and/or fragility of the sensor during manual labor and wanted a more rugged/waterproof design. Others ranked style and trendiness as a higher priority.

When discussing possibilities for long-term wear, participants reported that device comfort, ability to be integrated into their daily routine, and convenience were important aspects. Device material, size, and location were the most notable factors when discussing comfort. The wrist location of the sensor, tightness required for data collection, and relatively inflexible material limited range of motion in the wrist and was prohibitive for some activities. This was reported as particularly problematic for participants who worked in manual labor. Many participants reported that they integrated the sensor into their daily routine, which helped facilitate compliance; conversely, required breaks in sensor wear, including charging and showering, created opportunities for breaks in compliance (e.g., forgetting to put it back on). Finally, the data collection process used in this study (e.g., local data storage on the device, which required return visits to download) was unacceptable for more than a short course of monitoring; longer-term wear would require a more convenient data transfer process (e.g., Bluetooth transmission to a cloud-based server, or wireless transmission *via* a hub device).

Participants reported that bystander reactions were minimal and often mitigated by the assumption that the E4 was a fitness tracking device. Participants reported that the majority of family, friends, etc. were either indifferent or genuinely curious about the device but did not convey any negative perceptions. There was a notable difference in response from bystanders by age; younger people were more indifferent and less likely to even ask about the sensor, while older contacts were generally more curious. When the purpose of the device (and study) was disclosed to bystanders, many expressed interest in the concept of tracking medication, and specifically opioid use. The sensor was also questioned by non-study affiliated clinical personnel both in and out of the hospital; reactions ranged from curiosity to concern that the sensor would interfere with other clinical tests (i.e., magnetic resonance imaging or computed tomography scans). Clinical staff being unaware of the research protocol was considered a nuisance, and some participants reported this was particularly concerning to them.

Overall, the sensor was well-received by the majority of participants regardless of sex or opioid use history. Barriers to use varied depending on participant classification. Female participants more frequently cited size, lack of customizability, and aesthetics as barriers compared to males. Females were also more likely to find the device undesirably noticeable and to experience complications with wristband sizing. Multiple female participants compared the E4's looks to an “ankle bracelet,” referring to remote monitoring systems used in the criminal justice system. This specific comparison was not mentioned by any of the male participants. Device aesthetics were more likely to be mentioned as a barrier for participants within the opioid naïve class, while device mechanical specifications was the primary focus in the chronic and occasional classes. Specifically, some participants from the chronic and occasional classes reported being unsatisfied with the device's battery life or found charging the device every night to be excessive.

#### Change in behavior and thought process related to sensor use

The general consensus among participants was that wearing the sensor (and being monitored) did not change behavior significantly. They did note minor changes in daily routine (i.e., showering less because they did not want to take the sensor off), and a few people abstained from certain activities due to sensor-related restrictions in terms of movement and comfort. Some participants reported intentionally wearing long sleeves to conceal the device. None of these factors were cited as prohibitive but considered relatively minor annoyances. None of the participants felt that the sensor's presence changed the amount of opioid pain medication they took; however, several specifically mentioned that having the sensor on gave them the impression that they were being observed. In turn, they reported this perception made them more mindful of how many doses of opioids they ingested.

The majority of participants reported the sensor to have limited impact on their thought process. The participants that did report a perceived change were exclusively from the occasional opioid use class and described being more mindful/aware of the relationship between their opioid use and pain level (e.g., creating an opportunity to self-reflect and question whether the amount of pain they were experiencing truly necessitated prescribed opioids). Specific to the research, participants reported that this approach signified clinician commitment to conscientious opioid prescribing practices, which was viewed positively. Some participants in the chronic and occasional use subgroups also reported that using the sensor and participating in the study made them feel more prioritized and cared for by their clinical team. This increased sense of appreciation was not expressed among participants from the opioid naïve class.

#### Perceived usefulness in sensor-based monitoring

Many participants agreed that the application of sensors within the context of opioid use could be helpful but had difficulty verbalizing specific examples or use cases. When reflecting on their own experiences, the physical act of pressing a button/interacting with the device was most commonly cited as having some significance (i.e., by creating an increased awareness, or an opportunity to “pause” and think before taking a dose of pain medication instead of “just going through the motions”). In the context of opioid therapy, some participants thought it would be useful if the device could objectively determine the necessity for opioids, the presence or severity of pain, or both.

The concept of “othering” was recurrent in discussions surrounding prospective useful applications of the sensor. Many participants reported it would be helpful to detect opioid misuse and/or overdose in *others*, but none suggested that those functions would be useful (or needed) for themselves. Additional specific recommendations were made by the participants, but they again cited the target population of these applications being *others* [specifically though with OUD and/or mental health diagnoses, including post-traumatic stress disorder (PTSD) and anxiety].

Responses regarding device usefulness were more consistently obtained from opioid naïve participants than those from the occasional or chronic use classes, thus creating and unintentional sampling bias within this theme. Despite this limitation, opinions of sensor-based monitoring from chronic and occasional class participants were generally similar across opioid use class, and sex.

#### Willingness to be monitored and desired features in a sensor-based system

Participants reported willingness to continue being monitored/wear the sensor for a longer period of time, particularly in a research context. They often expressed that their motivation to do so was derived from a desire to help others and for the benefit of science. Most reported that home monitoring would also be acceptable if it benefited their care and likened it to other remote monitoring devices with which they have experience (i.e., home continuous positive airway pressure device for obstructive sleep apnea). When specifically probed, participants cited that an additional week to month would be the longest they would consider continuously wearing the current sensor for.

Although participants were willing to be monitored and wear the sensor for a longer period, they were less willing to wear two sensors at same time; this was perceived as “strange” and “excessive.” Several participants mentioned that although the sensor was easy to wear for a short period of time, there were some potential barriers to longer-term wear. This included work which prohibited such accessories and required manual labor that was either restricted by the senor or posed a perceived risk of damage to the sensor. Participants reported they would be willing to increase the duration of monitoring if the requested monitoring protocol was intermittent as opposed to continuous. Specifically, 24/7 monitoring was considered extreme and they wanted the option to remove the device while sleeping and performing certain activities.

Overall, willingness to continue would be greater if the study device incorporated more desirable features frequently found in commercially available devices participants already used. The two most commonly cited features were a clock face and fitness tracking capabilities. Participants wanted more interaction from device itself; only one expressed potential concern for notification fatigue. A companion phone app was more desirable than on-device notifications, and the types of notifications would have to be expected, targeted, and useful. Participants also wanted feedback in terms of device function (e.g., battery life), and their own progress towards goals (e.g., time elapsed since the last dose of their prescribed opioids, completed surveys). Some wanted the ability to see their data in real time: heart rate, step count, and glucose monitoring were all specifically mentioned. There were discrepancies in the amount of training that was considered acceptable or desirable, indicating a potential need for a personalized on-boarding process. Most participants thought the device (and any associated software) should be intuitive enough that training would be unnecessary. However, some participants who self-identified as having lower technology literacy thought that additional training would be desirable before they were expected to use the device on their own.

There were no differences in participants across sex and opioid use history classes related to the length of time they would be willing to wear the sensor beyond the study period. When asked if they would be willing to wear two sensors simultaneously (one on each wrist), subjects shared a similar degree of hesitation across both sexes. Of the three opioid use classifications, chronic opioid use class participants were found to be most willing to wear two sensors. In addition, female participants found the sensors rubber composition to be more irritating. Changing the composition of the sensor and the wristband to a more comfortable material, such as cloth or Velcro, was recommended to improve comfortability and sizing amongst multiple female participants. A similar minority of males and females suggested the addition of an interactive screen that could alert the user when it was time to take medication or complete a study task. Male participants specifically requested the addition of clock at minimum.

## Discussion

Overall, acceptance of the wearable sensor for opioid monitoring was high; participant perceptions were either positive or indifferent. Although participants wanted the device to look and feel like a commercial fitness tracker, they had different (and somewhat higher) expectations for a device related to medical monitoring. Specifically, they had lower tolerance for technical glitches or failures, and they expected other medical personnel to be familiar with it.

Although it was clearly explained that this was a novel research study, participants still had some basic expectations that other medical providers would be knowledgeable about the device and what it was doing. Clinicians not knowing about the device and/or study was disconcerting to participants, and sometimes led them to question its purpose and validity. Although this is expected (and impossible to overcome) during the initial research and development phases, consideration should be taken once interventions are more widely deployed in clinical practice. Widespread education to increase familiarity with other providers who are likely to be encountered would decrease the likelihood that the device gets questioned and provide additional reinforcement for it within the medical community.

Across the board, simplicity and intuitive features were highly desired. Most participants did not want “one more thing” to learn how to use; they preferred a plug and play-type system that you could simply turn it on and start using. However, some participants did explicitly request more training, specifically those with self-reported lower technology literacy and comfort. Intervention designers may consider varying levels of training based on some baseline assessment of technology literacy.

Othering is an interesting and telling phenomenon, whereby individuals isolate others based on some attribute (37). In this case, the “other” group that was repeatedly referenced were people who could progress to opioid misuse from a prescription. Participants acknowledged that this may happen to some people, but often considered it a risk to those who lack of self-control or willpower. The “othering” creates some questions about real world acceptance and usability if the purpose was for monitoring for misuse. Any stigma associated with a device-based intervention, for example the perception that the device is only indicated for patients that lack the will power or self-control to avoid opioid misuse on their own, will undoubtedly impact uptake. This should be very carefully considered during intervention design, education and deployment.

Some perception, barriers and facilitators differed among subgroups, and may be important considerations for intervention design. For example, opioid naïve participants more commonly cited aesthetics as a barrier, and were universally unwilling to wear multiple sensors. Individuals who were chronic opioid users (and thus generally expected to have more medical problems, and more routine interaction with the medical system) were more open to wearing multiple sensors and less concerned with aesthetics, but cited technical barriers (e.g., battery life), as important. The differences in daily life related to acute illness also had implications for sensor integration, both positive and negative. Pain, impairment, and the acuity of illness was in some cases distracting, and decreased willingness to learn something new and/or troubleshoot the sensor. Conversely, being acutely ill made participants less likely to engage in highly physical activities (including manual labor work), which was cited by some as a facilitator at least for short-term use. Considering the target user needs based on their stable individual characteristics and the dynamic circumstances surrounding their illness will be critical for usability and engagement.

### Limitations

Despite efforts to control for bias in the study protocol, there are still several potential sources of bias that should be considered when interpreting our results. Twenty seven percent of participants enrolled in the parent study did not complete the qualitative interview portion, and raises the potential for selection bias. There may have been systematic differences in preferences and perceptions between those who completed the study compared to those who did not, limiting our generalizability. The research staff was proficiently trained to facilitate participant interviews in a format that would limit bias by following a semi-structured interview guide without posing leading questions. However, as with all qualitative studies, the possibility for unintentional influence of a participant's response by an interviewer, such as through body language or tone of voice, cannot be discounted: the magnitude of this influence is not believed to be substantial. Finally, recall bias on the part of participants may have been exacerbated by severe levels of pain and/or mind altering effect of opioid analgesics taken while on study.

## Conclusions

In our sample of patients receiving prescription opioids in the inpatient and outpatient setting, acceptance of a wearable sensor for opioid use detection was high. Aesthetics, simplicity and seamless functioning were all reported as key to usability. The presence of the sensor did not appear to significantly alter participants' behavior. Perceived usefulness in monitoring was frequently stipulated by “othering” which should specifically be explored, and carefully considered during intervention design. The design of sensor systems targeted for opioid use must consider the physical, social and cognitive alterations inherent in the associated disease processed when compared to routine daily life.

## Data Availability

The datasets presented in this article are not readily available because Interview data is potentially identifiable and thus will not be made publicly available. Requests to access the datasets should be directed to stephanie.carreiro@umassmed.edu.
