# Supplementary material for: Perceptions on wearable sensor-based interventions for monitoring of opioid therapy: A qualitative study
Source: Front Digit Health. 2022 Oct 21;4:969642. doi: 10.3389/fdgth.2022.969642 (PMC9634745; doi:10.3389/fdgth.2022.969642)
Supplement: Supplementary file 1 [file Datasheet1.docx]

**Guide for Semi-Structured Qualitative Interviews**

1. **Section I: Perceptions on the use of the Biosensor**

**Intent:** The goal of this section is to explore subjects’ perceptions regarding the use of technology. Specifically, we will focus on use of the biosensor in terms of intrusiveness, usability, improvements, and future research.

1. Can you tell me about your experience with the sensor?
   1. Probe: What did you like about it? What didn't you like about it?
   2. Probe: Did you find it annoying or uncomfortable to wear? To use?
   3. Probe: Did you have any problems using the sensor? Tagging? Charging?
2. Is there anything you did differently because you were wearing the sensor?
   1. Probe: Anything you thought about differently?
3. Did anyone react to you wearing the biosensor?
   1. Probe: Hospital Staff? Family? Friends? Strangers?
   2. Probe: Who?
   3. Probe: What was their reaction? What did they ask any questions?
4. Would you be willing to wear the sensor for a longer period of time?
   1. Probe: How long?
   2. Probe: Why (or why not)?
   3. Would you ever consider wearing two sensors? One on each wrist?
5. **Section II: Future Research**

**Intent:** The goal of this section is to explore subjects' perceptions regarding future applications of the sensor technologies in the context of prescription opioid use.

- 1. If you needed an opioid medication in the future, would you use this device again to help monitor use/reaction to the medication?
  2. Do you think this would be a useful tool for doctors to monitor prescription opioid use?

1. **Section III: Subject Initiated Comments & Reflections**

**Intent:** The goal of this section is to allow subjects to provide any additional feedback.

1. Is there anything else that we did not cover that you would like to add?
   1. Probe: Anything about the biosensor?
   2. Probe: The About the study in general?
